# Surface Oxidation of Ethylenechlorotrifluoroethylene (ECTFE) Membrane for the Treatment of Real Produced Water by Membrane Distillation

**DOI:** 10.3390/ijerph15081561

**Published:** 2018-07-24

**Authors:** Zahra Anari, Arijit Sengupta, Sumith Ranil Wickramasinghe

**Affiliations:** Ralph E. Martin Department of Chemical Engineering, University of Arkansas, Fayetteville, AR 72701, USA; zanari@email.uark.edu (Z.A.); asengupt@uark.edu (A.S.)

**Keywords:** ECTFE membrane, surface property, produced water, fouling, membrane distillation

## Abstract

Modification of ethyleneechlorotrifluoroethylene (ECTFE) membranes by simple surface oxidation was reported in the present investigation in order to induce thin hydrophilic layer on hydrophobic membrane surface for the treatment of real produced water (PW). FTIR spectra indicates the appearance of hydrophilic functional groups (–OH and –COOH) on the membrane surface due to modification, while water contact angle, zeta potential measurement, EDX, XPS analysis confirmed the presence of O functionalized hydrophilic groups on the surface. The effect of modification temperature and the time of surface oxidation on the performance of the resulting membranes were studied systematically, which revealed that induction of optimized hydrophilicity can successfully reduce the organic fouling. However, too much hydrophilic surface induces polar/electrostatic interaction resulting salt deposition on membrane surface. A simple on site cleaning procedure was demonstrated to be successful for the treatment PW for at least three consecutive cycles of membrane distillation (MD).

## 1. Introduction

The water, trapped underground and brought to the surface during oil and gas exploration and production, was industrially termed as Produced water (PW) [1,2]. The physical and chemical properties of PW was found to be complex in nature and vary considerably depending on the location, geological formation and nature of hydrocarbons being produced. The salt content in produced water varies widely, from nearly freshwater to salt levels up to ten times higher than seawater [3,4,5]. A large variety of organic and inorganic compounds present naturally during formation, are transferred to the water through long-term contact with the hydrocarbon, or additives used during drilling and operation of the well can also be present in produced water. The presence of specific chemicals and the concentrations of those chemicals vary widely among different produced water samples. Some of the PW sample can have small concentrations of natural radioactivity. Produced water is one of the largest volume byproducts associated with oil and gas exploration and production. Approximately, 42,000 gallons of produced water are generated each year in the United States from about 900,000 wells [6,7]. Due to the government regulation pertaining to the environmental concern, the produced water cannot be disposed off into the environment without appropriate treatment. Therefore, proper management of the PW results a high cost (less than 1 cent/bbl to more than $5/bbl) for the oil and gas industries [2].

Membrane distillation is a thermally driven well established technique for the desalination of high TDS (total dissolved solid) water, in which hydrophobic porous membrane provides barrier for hydrophilic constituents present in the feed, allowing vapor to pass through and condensate in permeate side [8,9,10,11]. Ethylenechlorotrifluoroethylene (ECTFE) is one of the novel polymeric materials consists of alternating ethylene and chlorotrifluoroethylene units, finds importance for membrane based separation under harsh chemical conditions due to its excellent mechanical properties in a wide range of temperatures, and a wide variety of corrosive chemicals and organic solvents, including strong acids, chlorine, caustic solutions, strong polar solvents, and strong oxidizing agents [12,13]. Therefore, membrane distillation technique involving ECTFE membrane can be suitable for processing high TDS of PW. However, the organic content in PW can have detrimental effect in overall membrane performance due to organic fouling originated from strong hydrophobic interaction between hydrophobic ECTFE membrane surface and the organic constituents in the PW. Therefore, the surface properties of the membrane need to be modified in order to handle the situation.

Several approach was reported in literature on the modification of hydrophobic polyvinylidene fluoride (PVDF) and polypropylene (PP) membrane for the separation of oil-water emulsion [14,15,16,17,18,19]. Roughening of hydrophobic surfaces, followed by coating with low surface energy materials to develop super hydrophobic MD membranes can induce long-term wetting resistance and lower fouling propensity [17,20]. Though omniphobic MD membranes provide excellent wetting characteristics by repelling both water and oil, non-polar contaminants, having ultralow surface energy can be attracted to omniphobic surfaces via attractive hydrophobic–hydrophobic interactions [21,22,23]. In corporation of zwitterionic character on membrane surface was also found to be good for oil-water separation, but may not handle the high TDS feed solution [24].

Therefore, hydrophilic modification of the hydrophobic membrane surface can be suitable options in treating the PW samples [25,26,27]. The grafting of hydrophilic polymers on membrane surface can induce the hydrophilic characteristic, while too much hydrophilicity can lead to deposition of solid crystal of ionic compounds on membrane surface due to enhanced electrostatic interaction and can ultimately lead to wetting of the membrane. Moreover, grafting can lead to enhancement in boundary layer thickness resulting reduction in initial permeate flux. In the present investigation we propose simple surface oxidation of ECTFE membrane for the treatment of PW with improvement in initial permeate flux. The induction of extent of hydrophilicity can be tunned by optimizing the experimental conditions for the oxidation. A simple on-site cleaning procedure was also developed in order to demonstrate the reusability of the modified membranes.

## 2. Materials and Methods

### 2.1. Materials

ECTFE base membrane with 0.1 μm nominal pore size, 0.69 porosity and 91 μm thickness; was procured from MilliporeSigma (Billerica, MA, USA). Potassium permanganate (KMnO_4_) and nitric acid (HNO_3_) were procured from VWR, Radnor, PA, USA. Deionized water was used throughout the investigation was collected from Thermo Fisher 18 MΩ Barnstead Smart2Pure system, Schwerte, Germany. The produced water (PW) was collected from Southwestern Energy sites, Virginia after hydraulic fracturing and prefiltration. This collected PW was used throughout the investigation without any further pretreatment. Table 1 summarizes the characterization of PW.

### 2.2. Surface Modification

50 mL of de-ionized (DI) water was added into 3 g of potassium permanganate (KMnO_4_) to make 6.02 wt % KMnO_4_ aqueous solution. 3 mL of 64 wt % nitric acid (HNO_3_) was added into the purple potassium permanganate solution. The mixture was continuously stirred at room temperature for 30 min for complete homogenization. In the next step, the commercially available PTFE membranes were immersed into the KMnO_4_-HNO_3_ mixture. The surface oxidations of the membranes were allowed to continue in water bath for 40 and 60 °C for 1 and 2 h to achieve different extent of hydrophilicity on membrane surface due to the surface oxidation. After the desired reaction, the membranes were taken out and washed with DI water by ultrasonic cleaner for 30 min. Finally, the samples were dried and used for processing the PW and characterization.

### 2.3. Characterization

Fourier Transformed Infrared Spectroscopic (FTIR) analysis was carried out using IR Affinity, Shimadzu, Columbia, MD, USA; with a horizontal ZnSe accessary for wave length range of 700 to 4000 cm^−1^. A contact angle goniometer, Model 100, Rame-Hart Instrument Company, Netcong, NJ, USA; was employed to measure the contact angle in present investigation. Scanning electron microscopy (SEM) and energy-dispersive X-ray spectroscopy (EDX) analyses were carried out by Nova Nanolab 200 Duo-Beam, FEI, Hillsboro, OR, USA.

### 2.4. Membrane Distillation

For membrane distillation, 1 L feed was placed over a hot plate (60 °C) with continuous stirring. The 1 L distillate tank was placed in permeate side on a computer-connected balance (Mettler Toledo, Columbus, OH, USA) and was maintained at 20 °C using an external chiller (PolyScience, Niles, IL, USA). In order to monitor membrane wetting, break through or the quality of permeate, permeate conductivity was monitored continuously using a conductivity-meter, VWR, Radnor, PA, USA. A polycarbonate membrane module with 33.75 cm^2^ effective surface area was employed in present investigation. Polypropylene spacers (XN4510, Industrial Netting, Minneapolis, MN, USA) were used to provide mechanical support for the membrane and homogeneous contact of feed on membrane surface. Feed and permeate streams were allowed to pass in tangential mode with constant flow velocity using two peristaltic pumps (Masterflex I/P, Cole Parmer, Vernon Hills, IL, USA).

### 2.5. Regeneration

To regenerate the membranes after MD, 1 L of DI water was allowed to flush from both side at a constant velocity for 30 min. This simple cleaning procedure was proposed to be sufficient enough for onsite cleaning.

## 3. Results and Discussion

### 3.1. Surface Oxidation

**Scheme 1:**
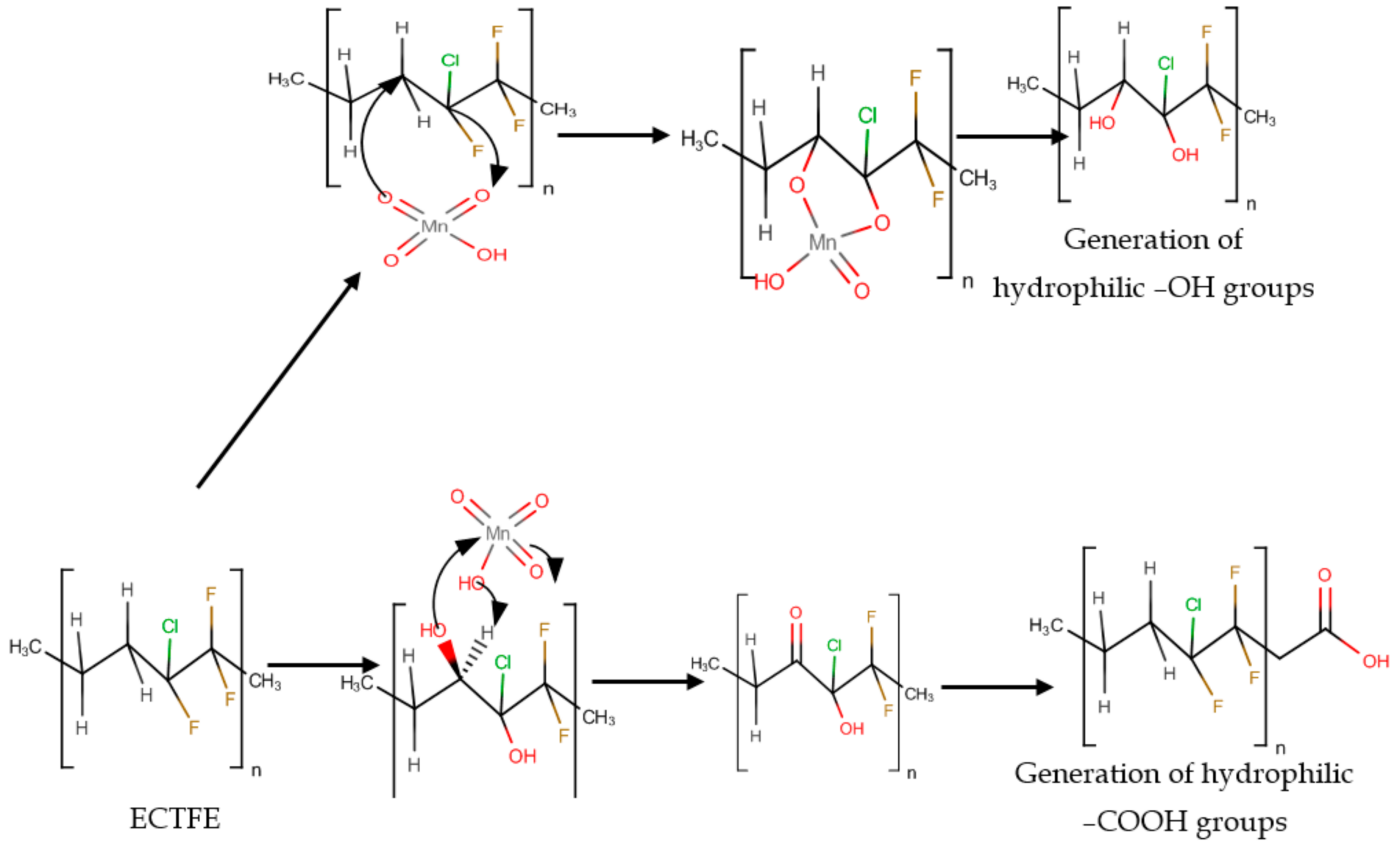
The schematic of the formation of hydrophilic functional groups (–OH and –COOH) on surface oxidation of ECTFE membrane by KMnO_4_.

The ECTFE membrane consisted of C–F, C–Cl and C–H bonds along with C–C backbone. Due to the absence of functional groups, the modification of ECTFE membrane surface is quite challenging. Mn in KMnO_4_ exists in +7 oxidation sate and is strong oxidant. In presence of strong oxidant like KMnO_4_, some of the above mentioned group can be oxidized along with the partial oxidation of C–C backbone [28]. As a consequence, presence of hydroxyl (–OH) and carboxyl (–COOH) groups were generated on the surface of the membrane Since the membrane surface was allowed to be in contact with KMnO_4_ solution, the surface oxidation is expected to be homogeneous. The presence of HNO_3_ helps in surface oxidation. High temperature reaction provides sufficient energy to the precursors in order to achieve the activation energy of the reaction enhancing the extent of surface oxidation at a particular time. As a consequence, the surface density of the hydrophilic group enhanced. On the other hand, the time of contact also improved the extent of surface oxidation resulting enhancement in hydrophilic groups on the ECTFE surface. The hydrophilic modification on hydrophobic ECTFE membrane is expected to reduce the membrane fouling from organic foulants present in the real produced water by reducing hydrophobic-hydrophobic interaction. However, presence of hydrophilic groups may induce favourable electrostatic interaction resulting scaling of inorganic salt on membrane surface during distillation operation. Therefore, it is required to optimize the hydrophilicity on membrane surface by applying different experimental conditions for surface oxidation. The formation of hydrophilic groups on surface oxidation of ECTFE membrane is presented schematically by Scheme 1. Longer duration of surface oxidation may start induce hydrophilicity at pore mouth exposure. In this case pore modification is quite unlikely, because the membrane material ECTFE is hydrophobic in nature and it is a prerequisite for membrane distillation membrane material. The surface oxidation reaction was carried out in aqueous medium. Therefore, probability of pore getting modified resulting inappropriate membrane distillation is negligibly small.

### 3.2. Characterization of the Modified Surface

#### 3.2.1. Water Contact Angle Measurement

**Figure 1:**
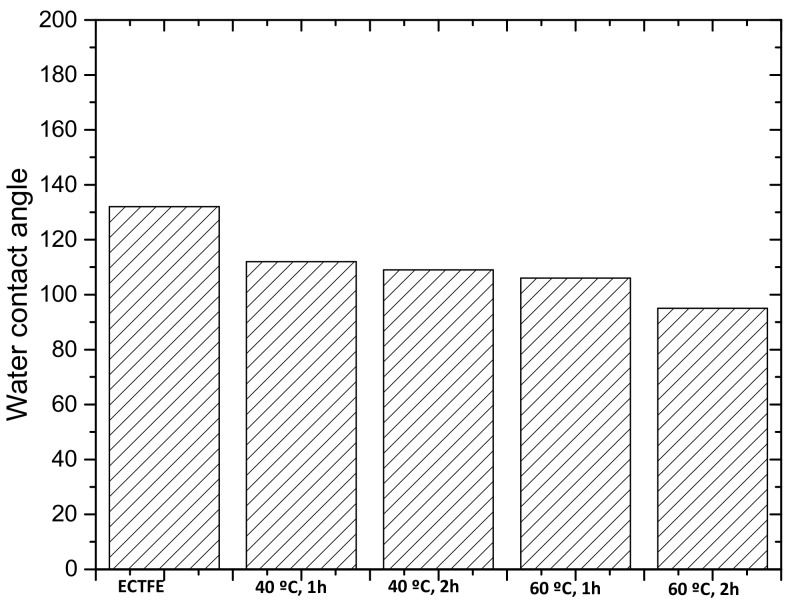
Water contact angles for modified ECTFE membranes.

Measurement of water indicated the hydrophilicity/hydrophobicity of the membrane surface [29,30,31]. Base ECTFE membrane showed the contact angle of 132, indicating the hydrophobic surface of the membrane, which is prerequisite for membrane distillation membrane materials in order to allow only water vapor to diffuse across the membrane, leaving the salt solution in the feed side. The surface oxidation at 40 °C for 1-h duration was found to induce hydrophilicity on membrane surface by reducing the contact angle to 112°, An enhancement in duration of surface oxidation did not show much reduction in water contact angle. This can be attributed to the fact that at 40 °C the overall reaction is quite slow compared to that at 60 °C. Therefore, to observe appreciable reduction in water contact angle at 40 °C, the duration of surface oxidation needs to be enhanced further. On the other hand, the water contact angles for the membranes modified at 60 °C were found to be lower than that of 40 °C due to more surface oxidation. It is also to be noted that, 3 h (or more) of surface oxidation at 60 °C makes the surface completely hydrophilic and is not worth anymore for membrane distillation operation. Figure 1 is summarizing the contact angles for the modified membranes.

#### 3.2.2. FTIR Characterization

**Figure 2:**
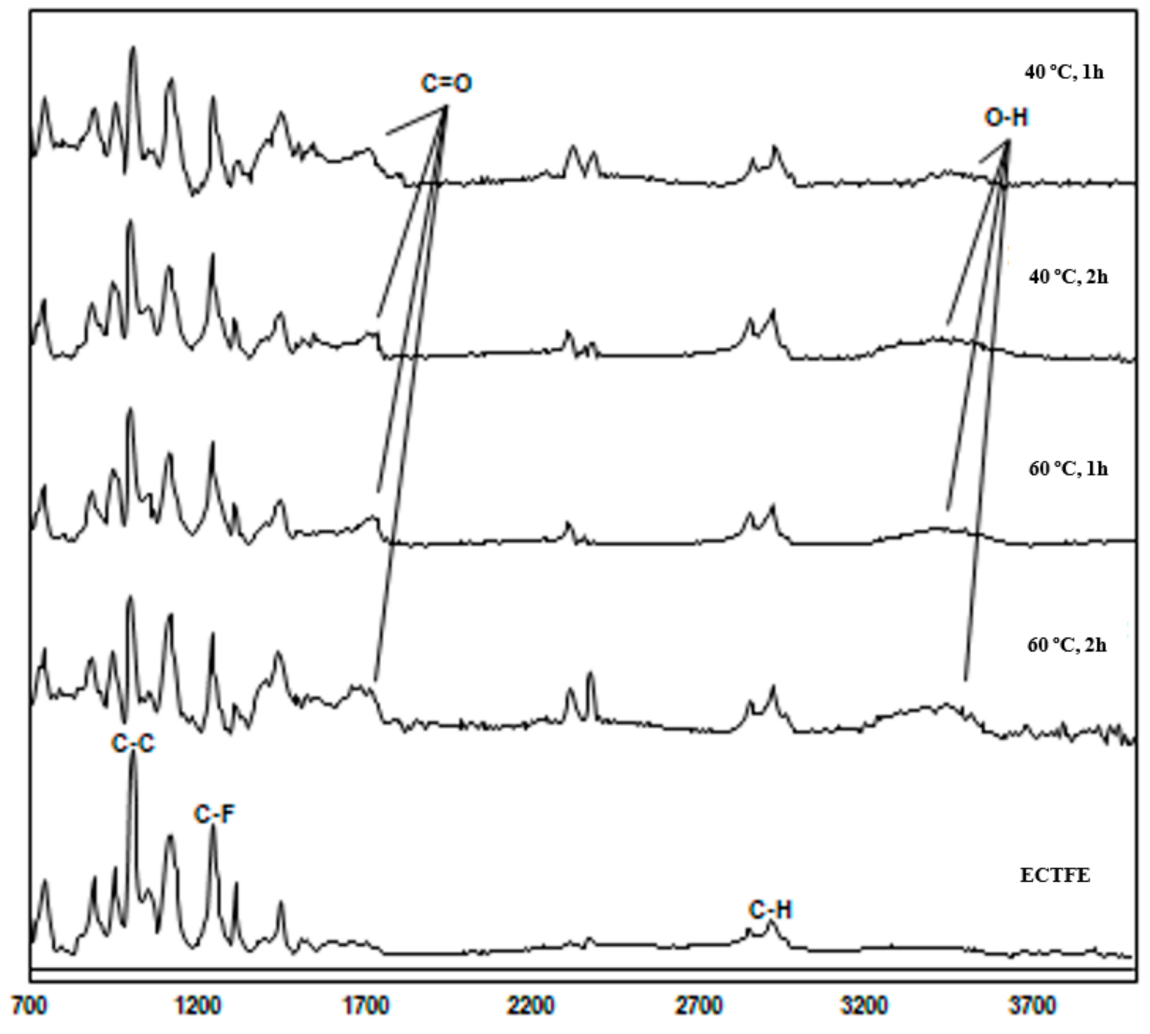
FTIR spectra for virgin and modified ECTFE membranes.

Figure 2 is showing the FTIR spectra for base and modified membranes. The FTIR spectra for base membrane showed signature peaks for C–C, C–H and C–F bonds, while for all the modified membranes, two additional prominent peaks were observed: one—1700 cm^−1^ and another broad peaks in the range of 3300–3500 cm^−1^ [1,32,33]. The first one corresponds to the carbonyl moieties (aldehylde, keto, carboxylic acid, ester, acid anhydride), whereas second one corresponds to the evidence of hydrogen bonded hydroxyl group. The FTIR clearly evidenced the surface oxidation of the ECTFE membrane resulting –OH and –COOH groups on the membrane surface. Though different experimental conditions should show different peak heights for the above mentioned peaks, but FTIR gives only the surface information of membrane up to 2 µm, due to its low penetration power. Hence FTIR peaks may not be used for quantitative purpose.

#### 3.2.3. EDX Characterization of Modified Membranes

**Figure 3:**
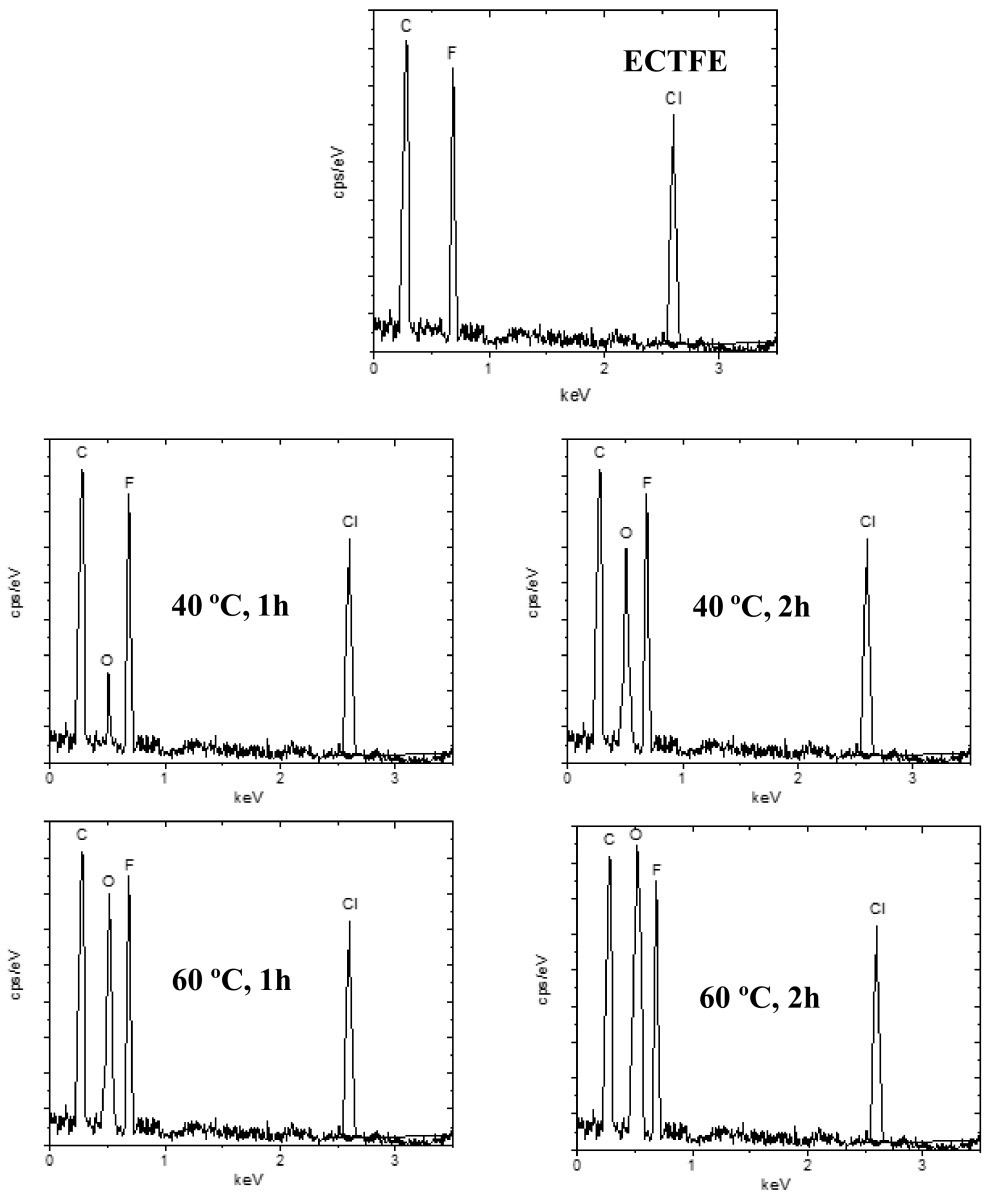
The EDX spectra for modified membranes.

Figure 3 is showing the EDX spectra for base ECTFE and the modified membranes. The base ECTF membranes showed prominent peaks −0.1 keV, 2.6 keV and 0.6 keV. These peaks correspond to C, Cl and F atoms, respectively [2]. On modification, an additional peak −0.5 keV was found to appear, which was attributed to the O. This implies the surface oxidation of ECTFE membranes to generate hydrophilic groups like –OH and –COOH. The extent of oxidation was also correlated by the intensity of the oxygen peak qualitatively. The membrane modified by 1 h at 40 °C was found to have oxygen peak of relatively lowest intensity, while that for 2 h and 60 °C was the highest. Though, EDX peak analysis is not quantitative in nature, however it gives an indication of extent of surface oxidation on ECTFE membrane.

#### 3.2.4. Zeta Potential Measurement

**Figure 4:**
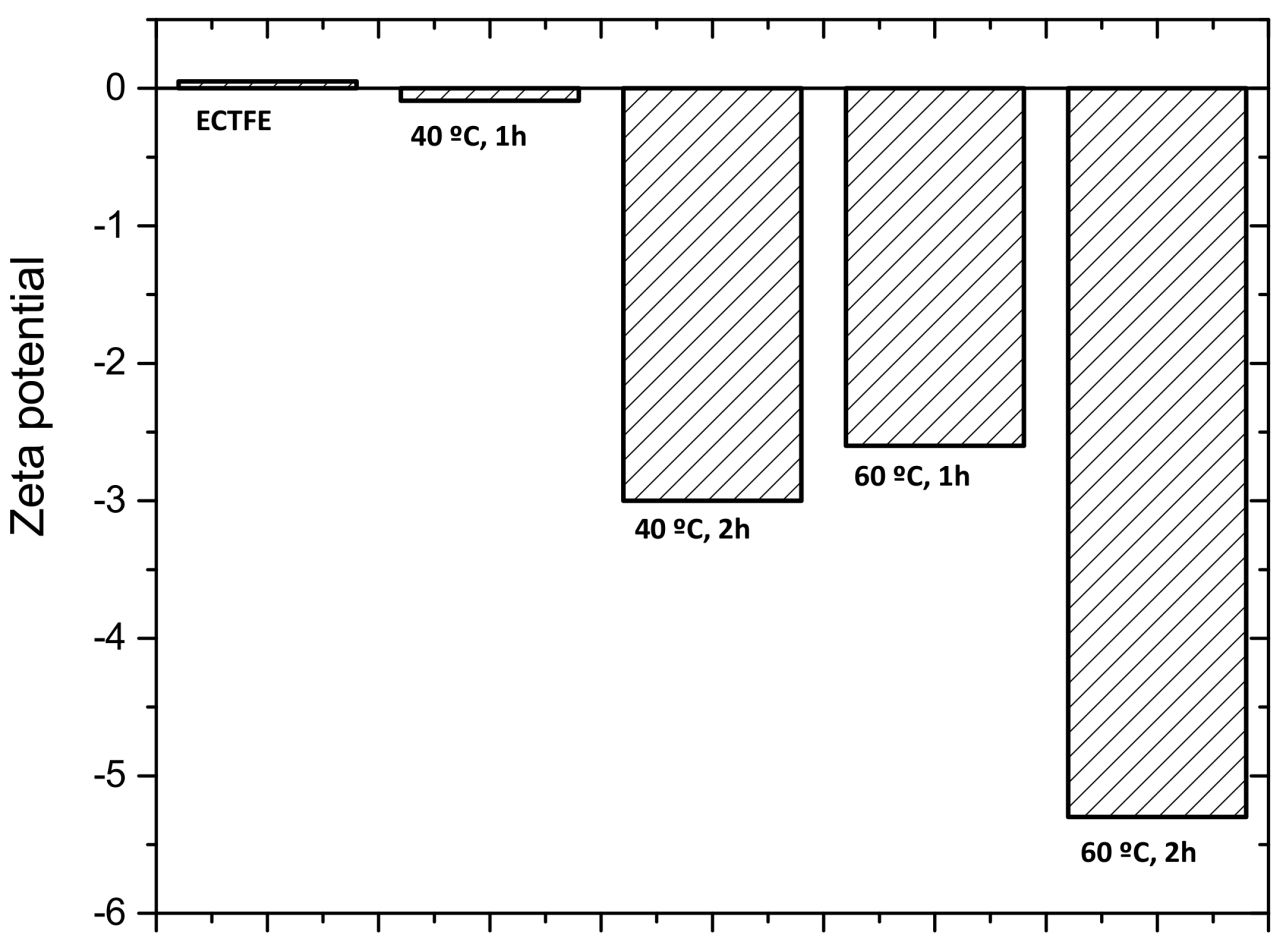
The zeta potential for the modified membranes.

Figure 4 is showing the zeta potential of the membrane after modification. The base ECTFE membrane was found to have almost no surface charge, which was quite expected as it has no functional groups on membrane surface and the membrane material is hydrophobic in nature. The membranes modified by 40 °C for 1 h did not show much change in zeta potential revealing the insufficient induction of hydrophilicity on membrane surface, while that with 60 °C 2 h showed a negative surface charge, indicating surface oxidation. The membranes modified with either 40 °C, 2 h or 60 °C, 1 h showed almost similar zeta potential, and hence their performance was expected to be similar.

#### 3.2.5. Relative Composition from XPS Analysis

Table 2 is showing the relative composition of C, F and O on modification from XPS analysis. The peak at 292 eV corresponds to –CF_2_-moieties, while C_1s_ peak was observed at 284 eV [34,35]. F_1s_ peak was observed at 686 eV, whereas O_1s_ peak was noticed at 529 eV [36]. The base ECTFE membrane did not show any O peak, while on modification the O peak intensity was found to enhance at the cost of reduction in relative composition of C and F peak. The membrane obtained from 60 °C, 2 h modification was found to have most intense O peak indicating induction of maximum amount of hydrophilicity on the membrane surface.

### 3.3. Membrane Performance

**Figure 5:**
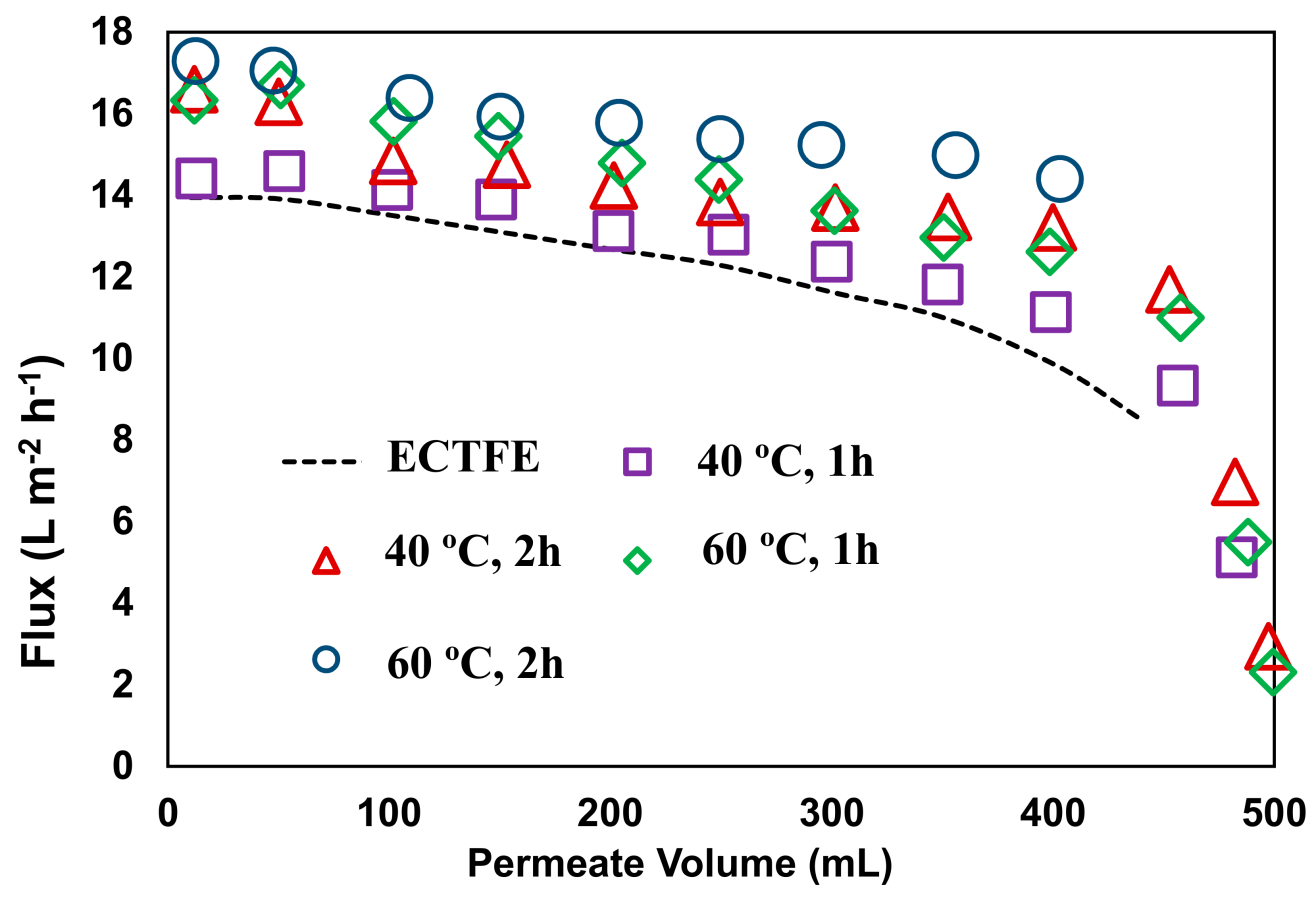
The performance of modified membranes during membrane distillation using real PW as feed.

From the surface characterization, it is decided to employ four kinds of modified membranes as follows: 1 h, 40 °C, 2 h 40 °C, 1 h 60 °C and 2 h 60 °C. The performances of these membranes were shown in Figure 5. The base ECTFE membrane showed an initial flux value ~14 LMH. With increase in permeate volume a drastic reduction in permeate flux was observed. This was attributed to the significant fouling from the organic substance present in the real produced water in terms of TOC. This membrane can only sustain up to a permeate volume of 450 mL, followed by wetting of the membrane. The modified membrane by 1 h and 40 °C showed only a marginal or no improvement in the membrane performance. This implies that the hydrophilicity is not sufficiently induced on the membrane surface in order to avoid fouling from organic matter. A gradual decrease in water flux up to 480 mL permeate volume was observed followed by drastic drop in permeate flux from 10 LMH to 6 LMH. The other modified membrane showed higher initial permeate flux compared to virgin membrane might be due to reduction in membrane thickness due to surface oxidation. Membranes modified by 2 h at 40 °C and 1 h 60 °C showed almost similar performance. Moderately steady flux values were observed for both of them up to 450 mL permeate volume with a flux value-12 LMH followed by drastic reduction in flux values. Ultimately, the flux values for these two membranes approached to zero and they were used for another two consecutive MD cycles after onsite suitable cleaning procedure developed in present investigation. These two membranes were shown to perform better than other membranes obtained by different experimental conditions. Membrane modified by 60 °C, 2 h got damaged around 400 mL permeate volume due to a very thin layer and hydrophilicity.

The polar organics, e.g., DMSO, MeOH, DMF acetone etc. would certainly interact with membrane surface leading to membrane fouling. However, for real PW, high molecular weight non-polar oily organic matters are of major concern and hence emphasized in present investigation.

### 3.4. Reusability Test

**Figure 6:**
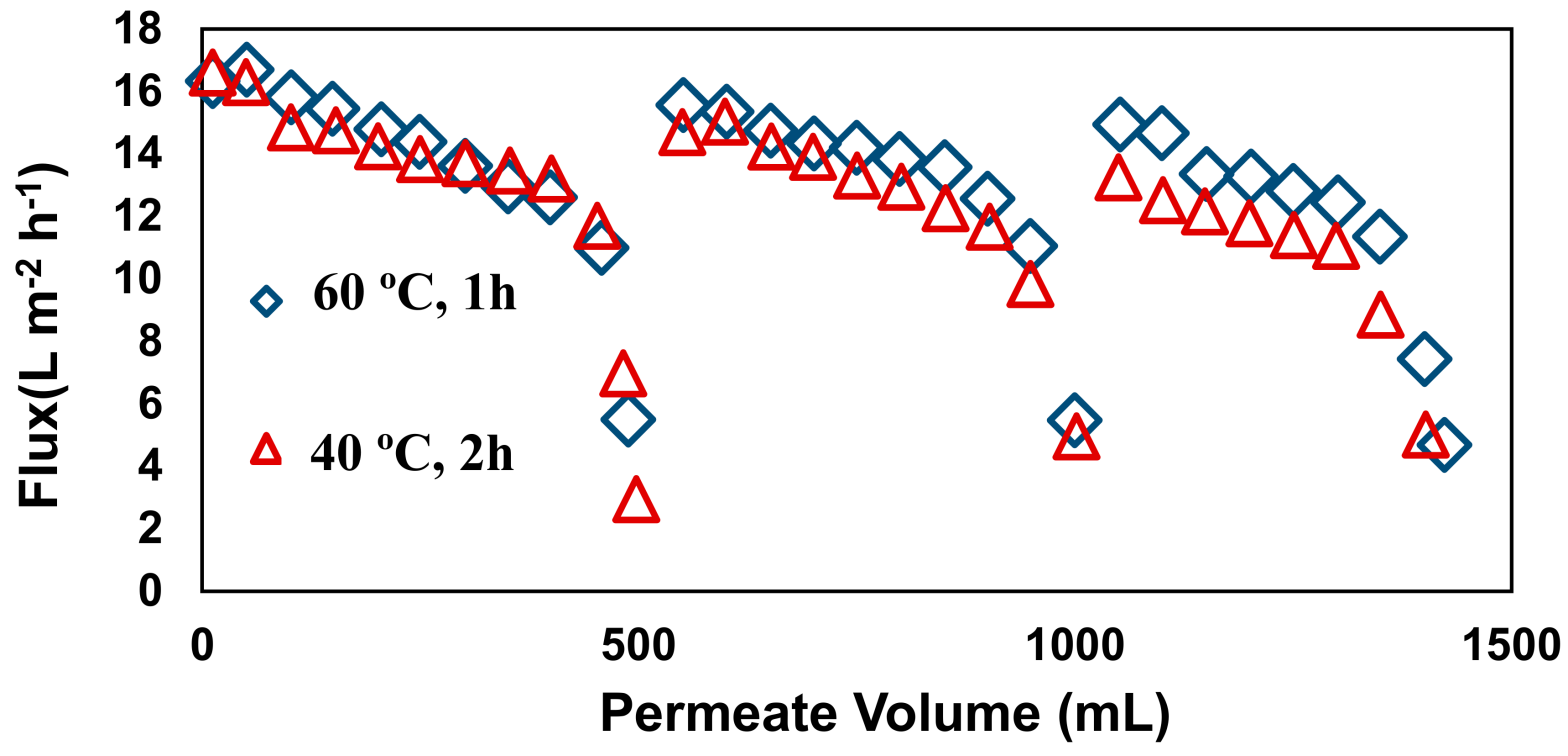
The performance of modified membranes after three consecutive MD cycles with onsite cleaning.

A simple onsite cleaning procedure was employed by flushing both feed and permeate side by DI water was found to be successful for removal of the foulant from membrane surface after MD operation. The reusability of the modified membranes has been tested for total three consecutive MD cycles. Almost 500 mL of permeate volume was found to be recovered at each MD cycle. The initial flux recovery for each MD cycle was found to be more than 90% suggesting the effectiveness of the cleaning method. The sudden drastic reduction of permeate flux at the end can be attributed to the deposition of inorganic salt on the membrane surface due to electrostatic or polar interaction between constituent ions in the PW feed and polar membrane surface. The performance of modified membranes in three consecutive MD cycles with onsite cleaning was shown in Figure 6. As seen from Figure 6, the membranes obtained from optimized reaction conditions (40 °C, 2 h and 60 °C, 1 h) were subjected to 3 consecutive MD cycles with onsite cleaning method in between. Both these membranes were performed successfully without any compromise on overall MD performance.

### 3.5. Membrane Fouling

**Figure 7:**
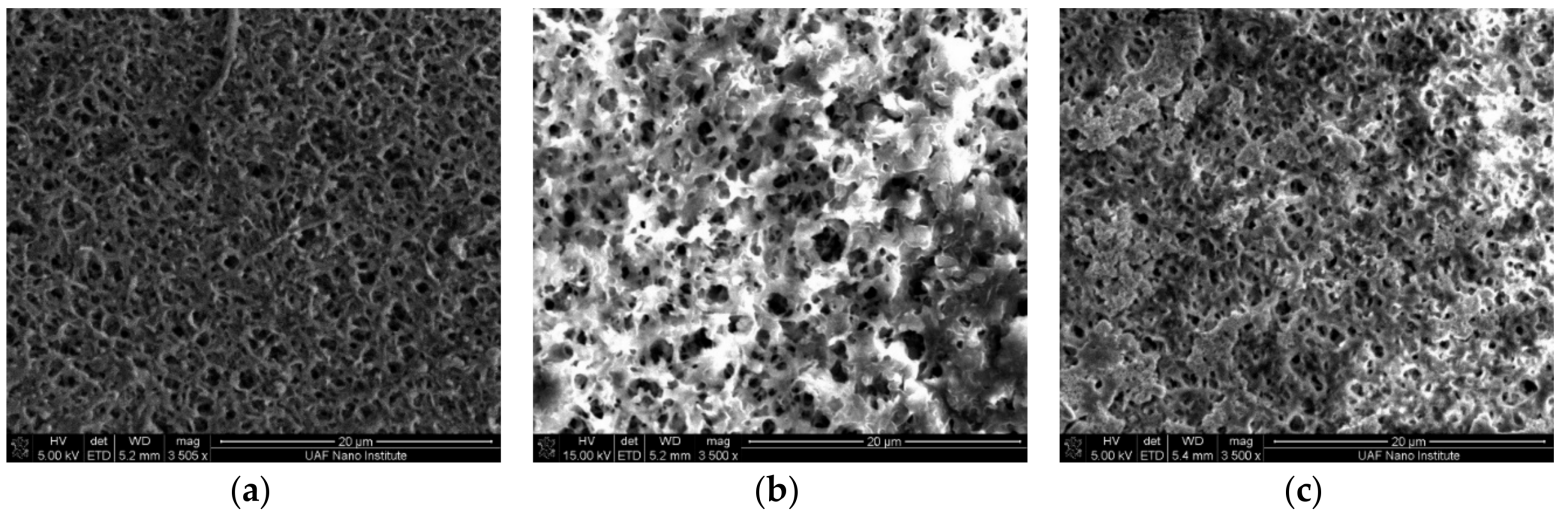

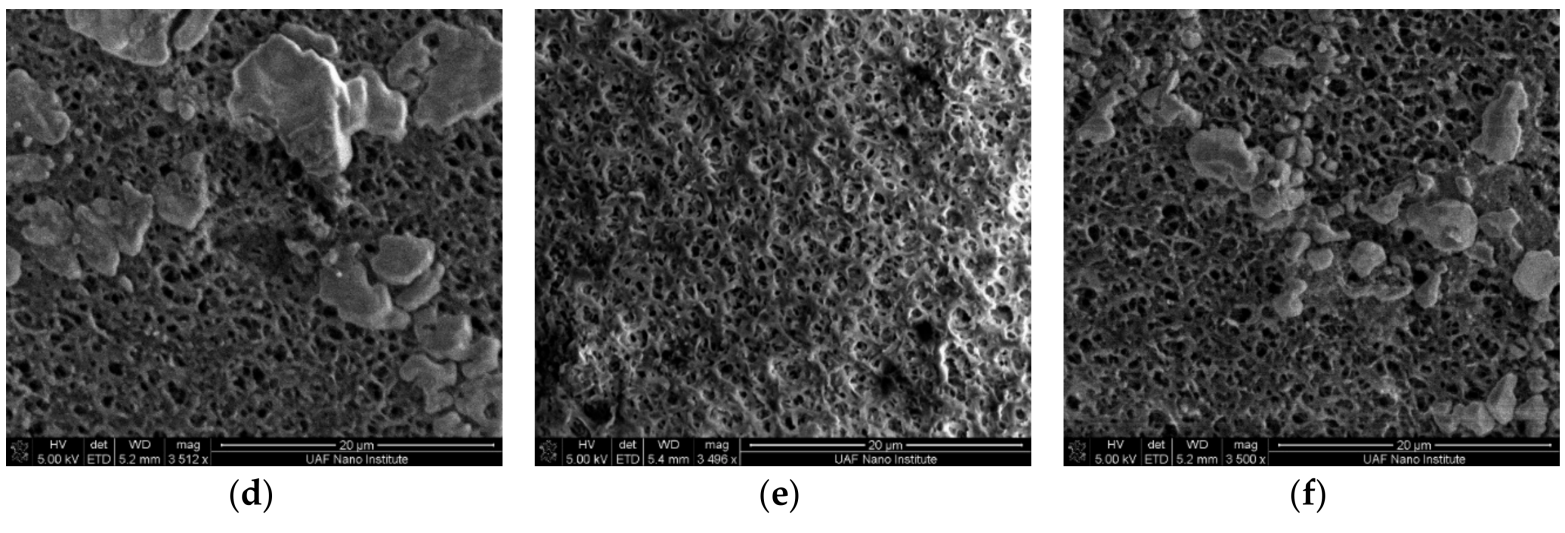
The SEM images for the fouled membranes (**a**) Base ECTFE; (**b**) base membrane after MD; (**c**) 1 h 40 °C modified membrane after MD; (**d**) 2 h 40 °C modified membrane after MD; (**e**) 1 h 60 °C modified membrane after MD; (**f**) 2 h 60 °C modified membrane after MD.

Figure 7 is showing the SEM images of the fouled modified membranes and its comparison with the base ECTFE membrane. After MD, the base ECTFE membrane was found to be fouled drastically and almost all of the pores on the membrane surface were found to be blocked [37,38]. The membranes modified by 2 h of surface oxidation (both 40 °C and 60 °C) showed deposition of solid crystal like species on the membrane surface and was attributed to the deposition of inorganic salt due to electrostatic interaction. On the other hand, the membranes modified for 1 h showed partial blockage of pores. However, the fouling was found to be more severe on base ECTFE membrane compared to any of the modified membranes. This SEM analysis was found to be in agreement with the MD performance of the membranes discussed in earlier sections.

**Figure 8:**
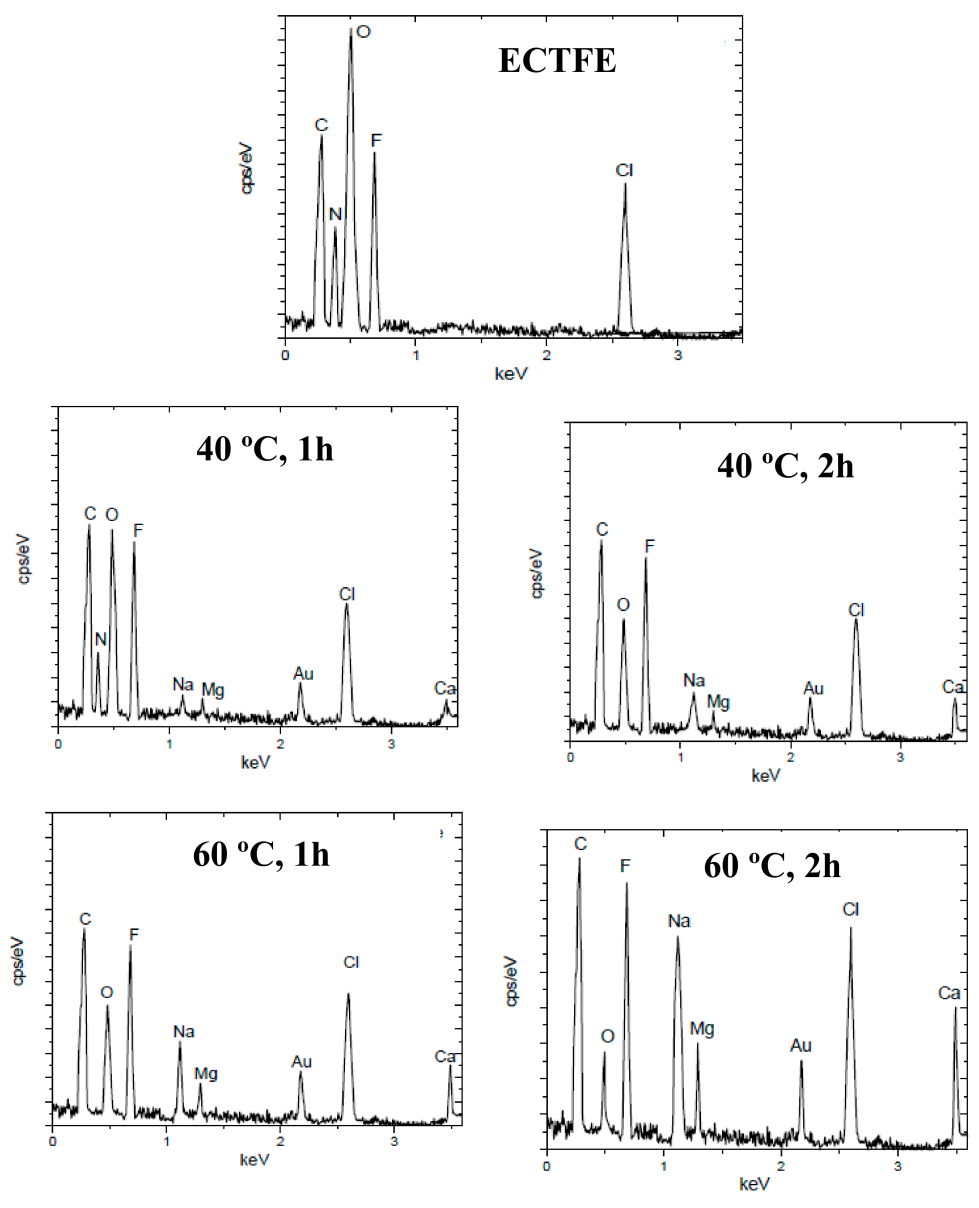
The EDX spectra for fouled membranes: base and modified.

Figure 8 is showing the EDX analysis of the fouled membrane. The EDX spectra of fouled base membrane showed enhancement in the intensity of O peak and appearance of N peak. This was attributed to the adsorbed organic compound on membrane surface as organic foulants [39,40]. The membrane modified by 1 h at 40 °C also showed similar peaks with the appearance of low intense Na, Mg and Ca peaks. This can be attributed to the fact that the extent of hydrophilicity on membrane surface is too low to avoid organic fouling but it also suffers a little bit from inorganic salt deposition due to electrostatic interaction. The membrane modified by 2 h at 60 °C showed large peaks for Na, Mg, Ca, revealing inorganic salt deposition. The membranes modified by other two experimental conditions (2 h, 40 °C and 1 h 60 °C) showed in between characteristics, i.e., significant reduction of organic fouling while appearance of little salt deposition on membrane surface. Thus tuning the experimental condition, required amount of hydrophilicity can be induced to the membrane surface in order to process high TDS as well as high TOC.

**Figure 9:**
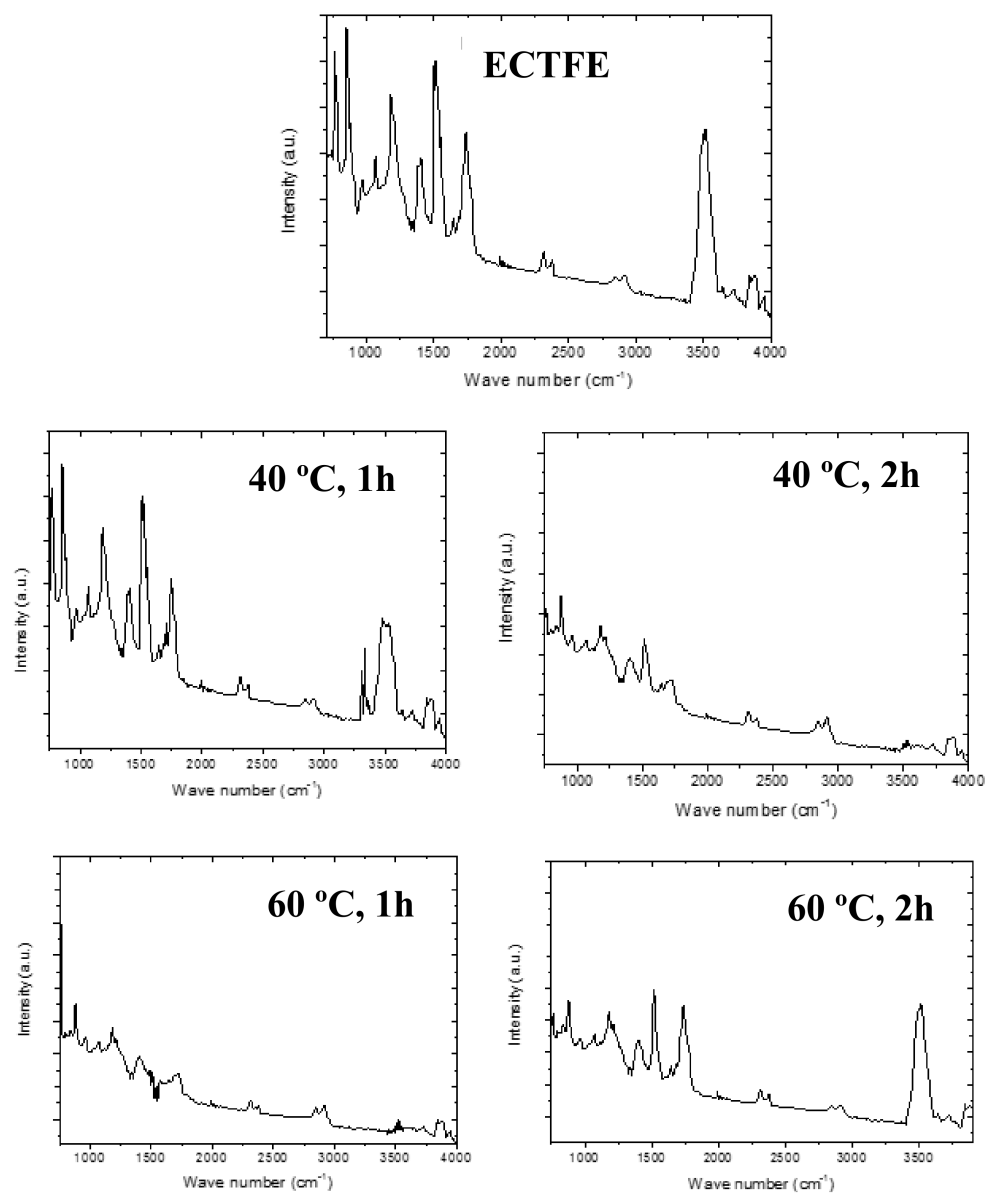
FTIR spectra for fouled membranes: base and modified.

Figure 9 is showing the FTIR spectra for fouled membranes; base and modified. The spectra clearly demonstrated high fouling on the surface of fouled base membrane. The appearance of stronger peaks—3500 cm^−1^ and 1735 cm^−1^ can be attributed to –OH/–NH bond and –C=O bond, respectively. This can be a polar end (exposed out of the membrane surface) of long chain organic compounds attached on membrane surface due to hydrophobic interactions. The overall surface functionalities got modified as seen from FTIR spectra. The membrane surface modified by oxidation for 2 h at 60 °C, showed predominance presence of –OH and –C=O functionalities due to fouling. Membranes modified by 40 °C, 2 h and 60 °C 1 h showed less fouling after MD experiments.

## 4. Conclusions

A simple surface oxidation of ECTFE membrane was found to be suitable for the processing of produced water by membrane distillation. The MD operation is useful for TDS removal, while the hydrophilicity induced by surface oxidation is useful in reduction of organic fouling. However, optimized hydrophilicity without enhancement of membrane thickness is better for processing real produced water without compromise on other membrane performance. Suitable choice of oxidation time and oxidation temperature can tune the extent of hydrophilicity on hydrophobic ECTFE surface. The surface oxidation leads to reduction in membrane thickness and thereby enhanced the initial permeate flux. The SEM image revealed the significant reduction in organic fouling for modified membrane, however the deposition of salt crystal cannot be avoided. A suitable onsite cleaning procedure was found to be effective and the reusability of these modified membranes was demonstrated for three consecutive cycles without much compromise in overall membrane performance.

## Figures and Tables

**Table 1:** Characterization of produced water.

Parameter	PW
Total dissolved solid (TDS)	157,000 mgL^−1^
Total suspended solid (TSS)	1300 mg L^−1^
Total organic carbon (TOC)	12 mg L^−1^
Turbidity	273 NTU’s
Calcium	12,000 mg L^−1^
Chloride	102,200 mg L^−1^
Magnesium	1250 mg L^−1^
Sodium	38,800 mg L^−1^
Sulfate	75.5 mg L^−1^
Electroneutrality percent difference	<5%

**Table 2:** The relative composition of C, O, F from XPS analysis.

Membrane	C (%)	F (%)	O (%)
ECTFE	64	36	-
40 °C, 1 h	61	32	7
40 °C, 2 h	52	24	24
60 °C, 1 h	56	21	23
60 °C, 2 h	51	20	29

## Abbreviations

ECTFE	Ethylenechlorotrifluoroethylene
PW	Produced Water
MD	Membrane Distillation
SEM	Scanning Electron Microscopy
bbl	Barrel
TDS	Total Dissolved Solid
PVDF	Polyvinylidene Fluoride
PP	Polypropylene
DI	De Ionized
FTIR	Fourier Transformed Infrared
EDX	Energy-Dispersive X-ray Spectroscopy
LMH	L m^−2^ h^−1^
